# Influencing Factors on the Physicochemical Characteristics of Tea Polysaccharides

**DOI:** 10.3390/molecules26113457

**Published:** 2021-06-07

**Authors:** Ting Hu, Peng Wu, Jianfeng Zhan, Weixin Wang, Junfeng Shen, Chi-Tang Ho, Shiming Li

**Affiliations:** 1 Hubei Key Laboratory of Economic Forest Germplasm Improvement and Resources Comprehensive Utilization, Huanggang Normal University, Huanggang 438000, China; wupeng201412@163.com (P.W.); zhanjianfeng2010@163.com (J.Z.); wangweixin1009@aliyun.com (W.W.); jfshen@hgnu.edu.cn (J.S.); 2 Fisheries Research Institute of Fujian, Xiamen 361013, China; 3 Department of Food Science, Rutgers University, New Brunswick, NJ 08901, USA; ctho@sebs.rutgers.edu

**Keywords:** tea polysaccharides (TPSs), chemical structure, tea species, processing technologies, isolation methods

## Abstract

Tea polysaccharides (TPSs) are one of the main bioactive constituents of tea with various biological activities such as hypoglycemic effect, antioxidant, antitumor, and immunomodulatory. The bioactivities of TPSs are directly associated with their structures such as chemical composition, molecular weight, glycosidic linkages, and conformation among others. To study the relationship between the structures of TPSs and their bioactivities, it is essential to elucidate the structure of TPSs, particularly the fine structures. Due to the vast variation nature of monosaccharide units and their connections, the structure of TPSs is extremely complex, which is also affected by several major factors including tea species, processing technologies of tea and isolation methods of TPSs. As a result of the complexity, there are few studies on their fine structures and chain conformation. In the present review, we aim to provide a detailed summary of the multiple factors influencing the characteristics of TPS chemical structures such as variations of tea species, degree of fermentation, and preparation methods among others as well as their applications. The main aspects of understanding the structural difference of TPSs and influencing factors are to assist the study of the structure and bioactivity relationship and ultimately, to control the production of the targeted TPSs with the most desired biological activity.

## 1. Introduction

Tea, an important agricultural product made from the fresh leaves and buds of plant *Camellia sinensis*, is the most consumed functional beverage in the world [1]. It has a long history of dietary and medicinal application, especially in Asian countries—such as China, Japan, India, and Thailand—for more than five thousand years [2]. According to the manufacturing process, tea can be categorized as unfermented green, white, and yellow teas, partly-fermented oolong and raw pu-erh teas, fully fermented black, pu-erh and dark teas, and post-fermented dark tea [3]. The processing methods including withering, rolling, fermentation, post-fermentation, and roasting of tea, and other factors such as cultivars, degree of ripeness, geographical location and agricultural practices will affect the content and structure of active compounds, resulting in the changes of biological activities. Tea possesses multiple biological functions, including antioxidant, hypoglycemic effect, anti-microbial, lowering blood lipids, and anticancer [1,4,5]. These biological activities have been attributed to the variety of chemical ingredients of tea, mostly to tea polyphenols such as catechins, theaflavins, thearubigins, theasinensins and other flavonoids, but also polysaccharides, alkaloids (caffeine, theobromine and theophylline), proteins, lipids, and inorganic elements (selenium, iron, manganese, etc.) among others [1,4,5,6].

**Figure 1:**
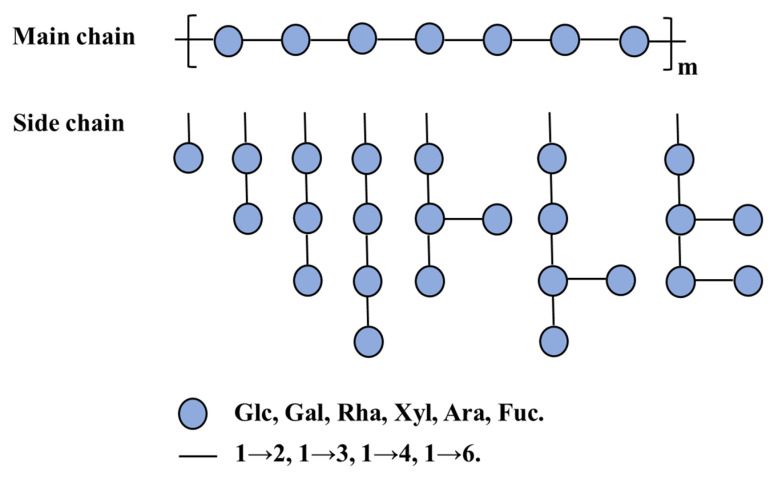
General structure of TPSs.

Other than tea polyphenols, the group of tea polysaccharides (TPSs) is one of the main bioactive constituents of tea and the content varies from approximately 1.5% to nearly 13% [7]. Figure 1 illustrates a general structure of TPSs, but is noninclusive due to the complexity of TPS structures. Recently, TPSs have attracted increasing levels of attention due to their various biological activities, including antioxidant [3,8,9,10,11,12], antitumor [13,14], anti-diabetes [12,15,16,17], antifatigue [18], anticoagulant [19], anti-obesity [20], hypoglycemic [16,21,22], and immunomodulatory activities [23,24]. According to the reports, TPSs contained 2–10 monosaccharides such as glucose (Glc), rhamnose (Rha), arabinose (Ara), mannose (Man), ribose (Rib), xylose (Xyl), galactose (Gal), fucose (Fuc), galacturonic acid (GalA), and glucuronic acid (GluA), and the monosaccharides linked by multiple glycosidic linkages such as 1→2, 1→3, 1→4, 1→6, leading to a wide range of molecular weight (M_w_) distribution (MWD) [4,7]. The molecular structure of a TPS is composed of multiple monosaccharide units. Similar to amino acids in proteins, the composition and linkage of individual monosaccharides have many different ways, resulting in variety of TPSs. Due to the nature of poly-monosaccharide unit composition, multiple sites of connection and chain conformation, the structures of TPSs vary dramatically in molecular weight, chain length and connection type, configuration and others. Hence the composition and connections of monosaccharide units are the defining parameters of TPSs. The molecular weight (M_w_) of a TPS is determined by the number of single monosaccharide units and the conformation of a TPS is directly associated with the types of monosaccharides and their linking position between two adjacent monosaccharides. Therefore, the components of monosaccharides and their connecting style are the basic foundations of TPSs. Factors affecting the composition and connection of monosaccharides are thereby influencing the structures of TPSs, which in turn influence the biological activities.

Therefore, to further study the biological property of TPSs and to effectively correlate the bioactivity and the structure of TPSs, it is also essential to understand and elucidate the fine structure of TPSs and the factors contributing to their variety, particularly the monosaccharide units and their connection. There are multiple factors that can cause the differentiation of monosaccharide composition and linkage, but the main ones are tea species, tea process, and isolation method of TPSs. The aim of this review is to provide a comprehensive summary of structural variety of TPSs and factors affecting the polymorphism of TPS structures, to associate the influential factors, TPS structures and bioactivity, and to enhance the characteristic understanding of TPS structure and to enhance the application of TPSs in the fields of bioactive polysaccharides and functional foods. 

## 2. Preparation of TPSs

**Figure 2:**
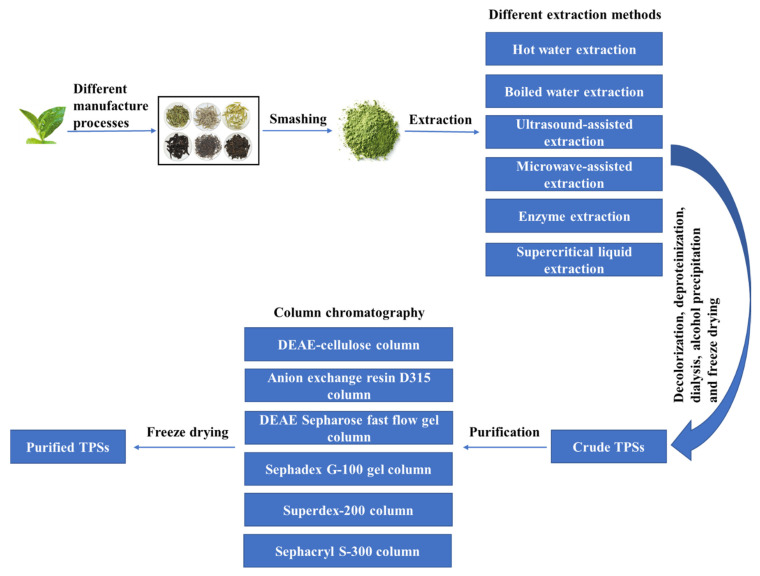
A schematic diagram of extraction and purification of TPSs.

A schematic diagram of extraction and purification of TPSs is shown in Figure 2. Different types of teas—including green, white, yellow, oolong, black, and dark tea—are obtained by different manufacture processes. Different extraction methods—such as hot water extraction (HWE), boiling water extraction (BWE), ultrasound-assisted extraction (UAE), microwave-assisted extraction (MAE), enzyme extraction (EE), and supercritical liquid extraction (SLE)—are used to extract TPSs from tea leaves, seeds, and flowers [9,16,25,26]. After decolorization, deproteinization and dialysis of the extracted solution, alcohol precipitation is used to obtain the crude TPSs. The crude TPSs are further purified by ultrafiltration or column chromatography filled with separating materials including DEAE-cellulose, Anion exchange resin D315, DEAE Sepharose, Sephadex G-100 gel, Superdex-200, Sephacryl S-300, and others [9,17,23,27,28,29]. The purified TPSs are usually lyophilized for readiness in characterization and biological activities study.

The traditional water extraction (WE) method is widely used because of its simple operation and low cost. However, due to its long extraction time and low yield, various assisted extraction methods such as UAE, MAE, and EE are developed to improve the yield of TPS. UAE and MAE have the advantages of simple, rapid, energy-saving and high efficiency, but they will have a certain impact on the bioactivity of TPS. The reaction conditions of EE are mild, which will not affect the bioactivity of TPS, but the cost is relatively high. In addition, a novel extraction method, SLE has the advantages of low energy consumption, high efficiency, mild, and environmental-friendly, but the equipment is expensive and the extraction time is long.

## 3. Physicochemical Characterization of TPSs

The physicochemical characterization of TPSs includes monosaccharide composition, molecular weight (M_w_), sequence of monosaccharides, location of glycosidic linkages, degree of branches, configuration, and conformation of the entire molecule. The monosaccharide composition of TPSs is usually analyzed using gas chromatography (GC) and GC-mass spectroscopy (GC-MS) after the hydrolysis of glycosidic linkages by trifluoroacetic acid (TFA) and derivatization with acetic anhydride. Gel permeation chromatography (GPC), gel-filtration chromatography (GFC), and/or multiangle laser light-scattering instrument (MLLS) are used to determine the M_w_ of TPSs. The chemical structures of TPSs are complex and determined by UV–vis, Fourier transform infrared spectroscopy (FT-IR), GC, GC-MS, 1D and 2D nuclear magnetic resonance spectroscopy (NMR), transmission electron microscopy (TEM), atomic force microscopy (AFM) combined with monosaccharide composition analysis, periodate oxidation, Smith degradation, partial acid hydrolysis, and methylation analysis. 

To date, more than 120 TPSs have been extracted and isolated from various types of tea. The main chemical characteristics of TPSs, such as the composition of monosaccharides, average M_w_ and chemical structure, are summarized in Table 1 and Table 2. As illustrated in Table 1, TPSs are heteropolysaccharides, consisting of 2–10 monosaccharides which contain Glc, Rha, Ara, Man, Rib, Xyl, Gal, Fuc, GalA, and GluA, with the average M_w_ ranged from 1.02 to 4940 kDa. There are only several studies about the chemical structure and chain conformation of TPSs, the main monosaccharides are Glc, Gal, Rha, Xyl, Ara, and Fuc and the linkages of main chain are 1→2, 1→3, 1→4, and 1→6 as shown in Figure 1. The conformation of TPSs in solution is characterized as sphere-like, random coil, and/or ordered helix-coil shapes [30,31]. The differences in monosaccharide composition, average M_w_ and chemical structure are closely related with the material, manufacture processes, extraction, and isolation methods of TPSs. 

The structures of TPSs vary due to different tea materials, harvest years, processing methods as well as extraction and purification methods among others. Table 1 and Table 2 summarized the factors including tea raw materials, processing technologies and isolation methods that affect the main chemical characteristics of TPSs, such as monosaccharide composition, M_w_, and chemical structure. 

### 3.1. Tea Material

The monosaccharide composition and M_w_ of TPSs differ from different parts of tea (i.e., leaves, flowers, seeds) and different species even in the same category of tea [10,11,40]. For instance, the M_w_ and monosaccharides of green tea TPSs were found different among species of Xihu Longjing, Huizhoulvcha, Chawentianxia, and others [11]. 

Tissues of tea—The leaves, flowers and seeds of tea had different profiles of monosaccharide composition and molar ratio of TPSs. Wang et al. analyzed the monosaccharide compositions of TPSs from tea leaves (TLPS), flowers (TFPS) and seeds (TSPS), and found that TLPS was composed of Gal, Fuc, Rha, Ara, Xyl, Man, Rib, GalA, and GlcA with a molar ratio of 1.00:0.29:0.87:1.27:1.77:0.07:0.11:0.3:2.54:0.24; TFPS was composed of Gal, Rha, Ara, Glc, Xyl, Man, GalA, and GlcA with a molar ratio of 1.00:0.42:0.97:0.36:0.11:0.17:0.71:0.08; and TSPS was consisted of Gal, Glc, Rha, Xyl, Ara, GalA, and GlcA with a molar ratio of 1.00:1.95:0.35:0.15:0.95:0.23:0.07 [10]. It was found that the in TPS from leaves had the most types of monosaccharides (nine) than that of TFPS (eight) and TSPS (seven). The molecular weight distribution (MWD) of TLPS, TFPS, and TSPS were ranged from 3.67 to 758 kDa, 2.56 to 1460 kDa, and 3.66 to 961 kDa, respectively, indicating that the M_w_ of polysaccharide from tea flowers was the highest [10]. In the same study, they also found that TFPS had higher M_w_ than TLPS [45].

Species of tea—TPSs extracted from different species of the same category of tea also had different monosaccharide composition and M_w_. For example, the monosaccharide composition of three species of green tea polysaccharides extracted from Xihu Longjing (XTPS), Chawentianxia (CTPS), and Huizhoulvcha (HTPS) was different. XTPS and CTPS were mainly consisted of Rha, Ara, Gal, Glc, Xyl, Man, and GalA with mole ratios of 9.50:8.79:13.17:5.52:1.24:3.52:11.60 and 8.73:11.95:16.53:11.06:1.17:3.81:15.06, respectively, whereas HTPS was consisted of Rha, Ara, Gal, Glc, Xyl, Man, Rib, and GalA with a mole ratio of 9.48:23.06:30.68:7.28:0.96:3.75:4.37:18.36 [11]. Compared to XTPS and CTPS, HTPS contained a new monosaccharide Rib, and the content of Ara, Gal, and GalA was the highest. The green tea polysaccharides from Shufeng, Longjin D, and Jialaoshan were composed of Rha, Ara, Xyl, Man, Gal, and Glu with different mole percentages of 3.15:33.90:3.36:2.45:38.44:18.70, 3.38:32.42:2.80:2.83:41.41:17.17, and 3.53:31.32:2.67:3.14:42.97:16.36, respectively [40]. TPSs from two types of Oolong tea, Fenhuangdanzong and Tieguanyin were composed of Rha, Ara, Xyl, Man, Gal, and Glu in the mole percentages of 3.65:31.70:2.79:2.60:44.87:14.38 and 5.77:32.56:3.81:1.22:46.47:10.18, respectively [40]. There was only difference in the mole percentages for three TPSs from green tea and two TPSs from Oolong tea.

The M_w_ of TPSs is also different among tea species. It was found that the M_w_ of TPSs from three green tea (Shufeng, Longjin, and Jialaoshan) and two oolong tea species (Fenghuangdanzong and Tieguanyin) were 127, 106, 121, 107, and 95, respectively. The M_w_ of polysaccharides from Tieguanyin oolong tea was the lowest [40]. Asides from the composition, the M_w_ of three green tea polysaccharides extracted from above mentioned XTPS, CTPS, and HTPS was also different as described in the following. XTPS was mainly consisted of three kinds of polysaccharides with the M_w_ of 810, 54.5, and 1.26 kDa, respectively; CTPS was mainly consisted of four kinds of polysaccharides with the M_w_ of 805, 138, 19, and 12 kDa, respectively; and HTPS was composed of four kinds of polysaccharides with the M_w_ of 771, 137, 11, and 1.2 kDa, respectively [11]. It was observed that the M_w_ of CTPS was generally higher than that of XTPS and HTPS in corresponding TPS range.

There is very limited research on the chemical structures of TPSs related to tea materials. In Scoparo’s study, the chemical structures of two kinds of polysaccharides from green (GSP) and black (BSP) teas were characterized and found that they both consisted of rhamnogalacturonan as the backbone containing a long sequence of →4)-6-O-Me-α-GalpA-(1→ and interrupted by α-L-Rhap residues. The difference was that GSP contained 65% GalA residues in comparison to only one third of GalA from BSP [35]. It is likely resulted from the oxidation during the processing of black tea, leading to the degradation of uronic acid.

### 3.2. Processing Technologies

Technology of tea process is another influencing factor on physicochemical characterization of TPSs. The monosaccharide composition and M_w_ can vary among TPSs extracting from teas with different processing procedures such as fermentation, aging, extrusion processing and selenium-rich technologies [8,12,28,43,50]. 

Fermentation—The composition and content of monosaccharides are different in the TPSs of unfermented green tea (GTPS), semifermented oolong tea (OTPS), and fermented black tea (BTPS). For example, the composition and molecular ratio of the main monosaccharides were found (i) GTPS: D-Rha, L-Ara, D-Xyl, D-Man, D-Gal, and D-Glc, with a ratio of 7.8:41.8:7.1:7.3:18.7:17.0; (ii) OTPS: D-Rha, L-Ara, D-Gal, and D-Glc, with a ratio of 16.2:43.7:18.0:21.9; and (iii) BTPS: D-Rha, L-Ara, D-Gal, and D-Glc, with a ratio of 14.4:36.4:19.7:29.4 [8]. It was found that OTPS and BTPS contained no D-Xyl and D-Man, suggesting fermentation had a significant impact on these two monosaccharides. The MWD of GTPS, OTPS, and BTPS was ranged from 9.2 to 251.5 kDa, from 5.3 to 100.9 kDa and from 3.8 to 32.7 kDa, respectively [8]. MWD was decreased with the increase of the fermentation degree, indicating that the glycosidic bonds in the backbone were cleaved during the fermentation process, and the larger degree of fermentation, the more cleavage. Moreover, the different degree of fermentation in the same category of oolong tea also resulted in discrepancies among corresponding TPSs. For instance, the polysaccharides from three oolong teas, namely, Tieguanyin (TTPS), Fenghuangdancong (FTPS), and Dahongpao (DTPS) with light, intermediate, and high degree of fermentation, respectively, were composed of mainly L-Rha, D-Fuc, L-Ara, D-Xyl, D-Man, D-Glc, and D-Gal with different molar ratios of 5.75:1.96:26.84:0.81:2.91:26.39:35.34, 10.83:3.83:25.69:2.39:6.97:14.44:35.85, and 10.31:5.09:22.93:0.28:5.21:22.59:33.59, respectively [50]. Therefore, the fermentation degree of tea effectively affected the molar ratio of monosaccharides of TPS, and the content of D-Xyl and D-Man decreased with the increasing of fermentation degree. Aside from that, the M_w_ of TTPS, FTPS, and DTPS was different: TTPS contained one major peak of 92.9% with M_w_ of 8.17 × 10^5^ Da and two minor peaks with M_w_ of 0.25 × 10^5^ (4.5%) and 0.07 × 10^5^ Da (2.7%); FTPS had two peaks with M_w_ of 9.30 × 10^5^ (34.2%) and 0.14 × 10^5^ (65.8%) Da; and DTPS consisted of one major peak 94.4% with M_w_ of 26.4 × 10^5^ Da and two minor peaks with M_w_ of 1.10 × 10^5^ (4.3%) and 0.42 × 10^5^ Da (1.4%), respectively. The largest M_w_ of oolong tea polysaccharides came from Dahongpao with the highest degree of fermentation [50]. The increased degree of fermentation decreased the M_w_ (or MWD), an opposite pattern from above mentioned TPSs from green, oolong, and black tea. Within the category of oolong tea, the reported data from the measurement of three oolong tea TPSs revealed that the increased M_w_ with the increased degree of fermentation, may result from three different species of tea prior to fermentation process, because the three brands of oolong tea in this example came from different regions of China. Species of tea plays more important roles in the composition and molecular weight than process in general.

Aging time—One of the characteristics of pu-erh tea is aging. Xu et al. (2014) studied the monosaccharide composition of polysaccharide isolated from pu-erh tea (PTPSs) with aging of mild fermentation for one year (PTPS-1), three years (PTPS-3), and five years (PTPS-5). The monosaccharide composition analysis showed that PTPS-1, PTPS-3, and PTPS-5 consisted of L-Rha, L-Ara, D-Xyl, D-Man, D-Gal, D-Glc, and D-Fuc with different molar ratios of 5.34:21.86:4.04:21.59:26.93:16.52:3.64, 6.82:26.22:0.35:13.83:39.34:10.23:3.21, and 15.98:20.84:0.15:15.29:40.33:6.08:1.68, respectively [12]. The monosaccharide composition of PTPS-1, PTPS-3, and PTPS-5 was the same, while the content of each monosaccharide was different. PTPS-1, PTPS-3, and PTPS-5 had different Mw. PTPS-1 had one major fraction of 92% with the M_w_ of 2.7 × 10^6^ Da; PTPS-3 had two major fractions with M_w_ of 6.31 × 10^5^ Da (52%) and 1.93 × 10^6^ Da (47%), respectively; PTPS-5 also had two major fractions with M_w_ of 1.16 × 10^6^ (60%) and 3.9 × 10^6^ Da (33%) Da, respectively [12]. Time of aging changed TPSs in different directions in terms of molecular weight, but more study was required to measure the molecular weight and amount of TPSs in the same time and then to correlate the MWD and the aging time, which could yield a conclusive pattern and reveal the relationship between aging or degree of fermentation with M_w_ in the comparison of same species.

Extrusion—The extrusion processing also influences the monosaccharide composition of polysaccharide from coarse tea [43]. Three kinds of TPS were extracted from untreated tea (TPSU), tea extruded at the conditions of 4% moisture content and 160 °C (TPSE4) and tea extruded at the conditions of 12% moisture content and 100 °C (TPSE12), respectively. The three kinds of polysaccharide samples were all composed of Rha, Ara, Man, Glc, Gal, and uronic acid. Extrusion treatment of the coarse tea resulted in the change of molar ratios on monosaccharide composition, which was 0.12:0.93:0.13:1.00:0.62:1.46, 0.20:1.11:0.18:1.00:0.63:1.23 and 0.88:1.19:0.34:1.00:1.00:1.93 for TPSE4, TPSE12, and TPSU, respectively. The MWD of TPSU, TPSE4, and TPSE12 was 0.1 × 10^4^ to 33.0 × 10^4^ Da, 1.5 × 10^4^ to 33.0 × 10^4^ Da and 0.4 × 10^4^ to 40.5 × 10^4^ Da, respectively, suggesting that extrusion treatment might destroy the cell structure and the high molecular weight polysaccharides were easily extracted [43].

Selenium enrichment—Selenium also has impact on the composition and structure of TPS. The monosaccharide composition of two Se-enriched tea polysaccharides (Se-TPS) extracted from artificial (ASe-TPS2) and natural Se-enriched teas (NSe-TPS2) were analyzed and found that ASe-TPS2 was composed of Rha, Ara, Glc, Xyl, and GalA in the molar ratio of 1.93:7.05:1.00:1.05:26.12, whereas NSe-TPS2 was consisted of Fuc, Rha, Ara, Gal, Glc, GlcA, and GalA in the molar ratio of 0.07:0.28:0.59:1.00:0.10:0.49:1.24 [28]. The main monosaccharide compositions of ASe-TPS2 were Ara and GalA, and the galacturonic acid was the highest among all five groups of monosacchrides. The main monosaccharides of NSe-TPS2 were Ara, Gal, GlcA, and GalA. The M_w_ of ASe-TPS2 and NSe-TPS2 was 6.73 × 10^3^ Da and 2.44 × 10^5^ Da, respectively, indicating that the M_w_ of Se-TPS from artificially Se-enriched green tea was smaller than that of Se-TPS from naturally Se-enriched green tea [28].

There are a few reports on the influence of processing technology on the chemical structure of TPSs. The selenylation method was found to affect the chain structures of TPSs [28]. The ASe-TPS2 extracted from artificially Se-enriched green tea was composed of β-D-(1→3)-Glcp, α-D-(1→4)-GalpA, (1→4)-Glcp, α-L-(1→2, 3)-Araf, α-L-(1→2)-Rhap, and α-D-(1→4)-GalpA, and non-reducing ends were consisted of Araf and Xylp, whereas the NSe-TPS2 extracted from naturally Se-enriched green tea was composed of β-D-(1→4)-Glcp and α-D-(1→4)-GalpA, and the branches were mainly composed of β-L-(1→2)-Araf, α-D-(1→3)-Galp, and β-L-(1→2)-Rhap, and the non-reducing ends were mainly composed of Glcp and Galp residues. The difference between ASe-TPS2 and NSe-TPS2 may be influenced by the Se element.

### 3.3. Isolation Methods

The monosaccharide composition, molar contents, M_w_ and chemical structures of TPSs are different under different isolation methods, which are listed in Table 1 and Table 2. 

Extraction methods—Tea polysaccharide conjugates can be extracted with water or alkali solution, containing neutral sugars, uronic acid, and protein. Water-soluble TPS conjugates (TPC-W) and alkali-soluble TPS conjugates (TPC-A) were extracted from green tea by hot water and alkali solution respectively [39]. The TPC-W and TPC-A were both composed of seven monosaccharides, namely Rha, Fuc, Ara, Xyl, Man, Glc, and Gal with different molar ratios of 8.74:4.69:29.04:0.42:7.11:14.10:35.89 and 13.81:1.43:36.07:5.24:4.89:6.28:32.27, respectively [39]. The difference in monosaccharide composition between them was not much, but there was a significant difference in molecular weight. TPC-W had three homogeneous components with the M_w_ of 6.62 × 10^3^ (56.07%), 4.85 × 10^4^ (6.54%), and 4.55 × 10^6^ (37.38%) Da, respectively; TPC-A was consisted of four homogeneous components with the M_w_ of 4.13 × 10^3^ (17.14%), 1.12 × 10^4^ (11.43%), 6.77 × 10^4^ (2.86%), and 4.94 × 10^6^ (68.57%) Da, respectively. Hence, the higher M_w_ of TPC was more efficiently extracted by alkali solution than water [39]. Wang, Yang, and Wei studied the monosaccharide composition of polysaccharides from leaves and flowers of green tea obtained by different extraction methods [45]. The polysaccharides from leaves with hot water (HWE-TLPS) and boiled water extraction (BWE-TLPS) were composed of Rha, Ara, Gal, Glu, Xyl, Man, GalA, and GluA with molar ratios of 4.82:0.22:2.93:1.00:0.21:0.48:2.06:0.22 and 0.50:1.04:1.38:1.00:0.06:0.22:1.48:0.09, respectively. The polysaccharide extracted from tea leaves with enzyme extraction method (EE-TLPS) was composed of Rha:Ara:Gal:Glu:GalA:GluA and the corresponding molar ratio was 1.09:1.80:2.27:1.00:2.36:0.12. Xyl and Man were not found in EE-TLPS, indicating that Xyl and Man were easily destroyed under the enzymatic extraction conditions. The polysaccharides extracted from tea flowers with hot water (HWE-TFPS), boiling water (BWE-TFPS), and enzyme extraction (EE-TFPS) were all composed of Rha, Ara, Gal, Glu, Xyl, Man, GalA, and GluA with different molar ratios of 0.36:1.19:2.09:1.00:0.16:0.23:0.25:0.12, 0.80:2.06:2.47:1.00:0.20:0.25:2.20:0.13, and 1.33:2.90:3.20:1.00:0.24:0.21:2.10:0.12, respectively [45]. The content of Rha, Ara, and Gal in EE-TFPS increased due to the effect of enzyme. HWE-TLPS and BWE-TLPS were mainly consisted of three kinds of monosaccharide components with the M_w_ of 1.165, 104, and 413 kDa (HWE-TLPS) and 1.04, 138, and 458 kDa (BWE-TLPS), respectively. EE-TLPS was mainly made up of five components with the molecular weight of 1.023, 49.3, 75.9, 151, and 487 kDa. HWE-TFPS, BWE-TFPS, and EE-TFPS were mainly consisted of four monosaccharides with the molecular weight of 483, 168, 120, and 1.059 kDa (HWE-TFPS); 508, 168, 120, and 1.059 kDa (BWE-TFPS); and 465, 157, 129, and 1.176 kDa (EE-TFPS), respectively [45]. It can be seen that the M_w_ of polysaccharides using enzyme extraction were decreased, indicating that enzyme catalyzed cleavage of TPS bonds occurred in the extraction process. Three tea flower polysaccharides (TFPS) were prepared by traditional water extraction (TWE-TFPS), microwave assisted extraction (MAE-TFPS), and ultrasound assisted extraction (UAE-TFPS), respectively. The peak M_w_ from TWE-TFPS was 4.4 and 31 kDa. Comparing with TWE-TFPS, the peak M_w_ from UAE-TFPS decreased with the increasing ultrasonic power, whereas the peak M_w_ from MAE-TFPS increased with the augment of microwave power, demonstrating that radio waves have significant effects on the M_w_ of TPS [56]. 

Purification methods—Isolation methods will also have dramatic influence on the M_w_ and chemical structures of TPSs (Table 2). In Gao’s study, the M_w_ of the TPS from five subfractions isolated and purified with different concentrations of NaCl aqueous solution (0, 0.1, 0.2, 0.3, and 0.4 M) from a local Chinese tea (Zhongcha #108) was 51.85, 40.0, 32.72, 25.27, and 18.38 kDa, respectively, and the M_w_ was decreased with the increase of the concentration of NaCl solution [5]. The stronger ionic strength of the solution yielded the lower Mw of TPS, indicating that the solubility of large TPS is lower than that of small TPS in strong ionic solution. From the crude tea polysaccharide (NTPS) of green tea, a neutral polysaccharide (NTPS-1) was extracted with plant hydrolase and precipitated by 95% aqueous ethanol, and NTPS-1 was a galactan consisting of β-(1→4)-linked galactopyranosyl units [55]. An acidic polysaccharide (ATPS-2) was extracted with cellulose compound enzyme and precipitated with 75% aqueous ethanol from green tea. The backbone of ATPS-2 was →4)-α-D-GalpA-(1→2)-α-L-Rhap-(1→4)-α-D-GalpA-(1→ and the side chains attached to the α-L-Rhap residues, which was quite different compared with NTPS-1 [29]. Two homogenous water-soluble polysaccharides (TPS1-2a and TPS1-2b) from green tea leaves were isolated from crude tea polysaccharides (TPS1) extracted with hot water and followed by 40% of aqueous ethanol precipitation after purification with gel permeation. TPS1-2a and TPS1-2b were homogalacturonan (HG) pectins consisting of (1→4)-α-D-GalA residues as backbone with 28.4% and 26.1% of carboxyl groups as methyl ester, respectively [23]. The investigation of the chemical structures of water soluble fractions (GTPS and BTPS) and insoluble fractions (GTPI and BTPI) from green and black teas found that soluble fractions GTPS and BTPS were consisted of a main chain of (1→3)-β-Galp with side chains of α-Araf and terminal units of α-Araf, α-Fucp and α-Rhap substituted at O-6 by (1→6)-linked-β-Galp, whereas water insoluble fractions, GTPI and BTPI were composed of a main chain of (1→4)-β-Xylp, substituted at O-3 by α-Araf, β-Galp, and α-Glcp units [54]. In another study, two polysaccharide fractions termed TFP-1 and TFP-2, were separated from Sephadex G-100 column chromatography from the crude polysaccharide (TFP), which was extracted from tea flower by boiling-water extraction and ethanol precipitation. It was found that TFP-1 contained α-L-Rhap, α-D-Galp, α-D-GalpNAc, α-D-Xylp, α-D-Glcp, and β-D-Glcp residues, whereas TFP-2 is composed of α-L-Rhap, α-L-Arap, α-D-Xylp, α-D-Glcp, and α-D-GlcpNAc residues [9], illustrating that the separation methods play an important role in the composition and structures of TPSs, reminiscent of small molecular separation.

Drying methods—Four polysaccharides were isolated from tea leaves using freeze-drying (TPS-F), vacuum-drying (TPS-V), spray-drying (TPS-S), and microwave-vacuum drying (TPS-M), respectively. These four crude tea polysaccharides were all composed of Rha, Rib, Ara, Glc, Xyl, Gal, Man, GalA, and GluA with different molecular ratios of 1.0:0.58:2.77:2.82:0.14:2.44:0.45:3.88:0.26, 1.0:0.47:2.62:5.96:0.16:3.43:0.51:3.0:0.20, 1.0:0.46:2.43:0.77:0.10:3.24:0.40:1.94:0.18, and 1.0:0.29:1.90:1.30:0.15:2.97:0.40:1.66:0.16, respectively [44]. The content of GalA and GluA in TPS-S and TPS-M were lower than TPS-F and TPS-V. TPS-F, TPS-V, TPS-S, and TPS-M showed similar molecular weight distribution and mainly contained four distinct peaks with groups of molecular weights around 9.2 × 10^5^, 2.2 × 10^5^, 3.0 × 10^4^, and 0.34 × 10^4^ Da, respectively [44]. The difference of monosaccharide ratio and MWD can be resulted from the different drying methods of TPSs. 

## 4. Applications of TPSs

TPSs have a variety of biological activities, such as antioxidant [3], hypoglycemia [15], anti-fatigue [18], anti-obesity [20], prebiotics [57], and immunomodulatory effect [24], it can be added to food as a functional ingredient to prepare health products. At the same time, TPS is a good emulsifier, which can be used in food, cosmetic and pharmaceutical industry. In Chen’s study, an alkali-extracted tea polysaccharide conjugates (TPC-A) was used to stabilize oil-in-water emulsions, and found TPC-A had a favorable protective effect on catechins and can be used as a natural emulsifier [58]. Li et al. (2021) also obtained a natural antioxidant emulsifier from Chin brick tea, tea polysaccharide conjugate (TPC) possessed a good emulsifying properties with excellent antioxidant activity, which can be used as dual-purpose antioxidant emulsifiers [59]. In addition, TPSs can be used as feed additives in the poultry and feed industry to enhance animal immunity and improve meat quality. For example, a green tea polysaccharide conjugates (GTPC) was extracted from *Yingshan Yunwu* tea could improve immune status, intestinal microflora and meat quality in chickens [60]. Moreover, in the biomedical industry, TPSs can be used as a drug delivery agent. In Li’s work, a biodegradable, non-toxic and environmental-friendly PTX loaded nanoparticle was prepared using TPS as the shell and zein as the core, it was found that TPS would be a promising agent in the drug delivery system [61]. Wu et al. (2018) also synthesized a cationic branched tea polysaccharide derivative (CTPSA), which exhibited lower cytotoxicity and can be used as a nonviral vector for the delivery of siRNA to the liver [62]. However, the application of TPSs are few or even a blank in other fields such as wound treatment, antiviral preparations, fertilizers, etc. which may be related with the complexity of TPSs structure and unclear structure-activity relationship of TPSs. Therefore, a lot of work needs to be done to develop the potential application of TPSs in the future.

## 5. Conclusions

The different structures of TPSs obtained in various reports are due to different tea raw materials, processing technologies, and isolation methods. The structural features of TPSs differ from different teas, even different parts and categories of the same tea. The structures of TPSs obtained with different degree of fermentation and extraction methods could have vast differences. Therefore, it is critical to clarify how the fermentation process affects the structure of TPSs and find an optimal method to obtain targeted TPSs with higher bioactivities. Due to the multiple influencing factors discussed in this review and the complex structures of TPSs, it is currently impossible to predict and also quite difficult to determine the structures of TPSs, especially high-level structures. Moreover, it is unrealistic to speculate the efficacy of TPSs without the characterization and actual biological testing of isolated TPSs. Thus, a large number of experiments are required to identify the complete structures of TPSs and to further evaluate the bioactivity of characterized TPSs. Furthermore, most of the researches focused on the polysaccharides from green tea, whereas the studies about polysaccharides from other teas are scarce. Therefore, more studies should be also focused on the characterization, identification, and comparison of the structures of TPSs from different teas with different processing technologies and isolation methods to have a comprehensive evaluation and understanding of the factors influencing the structures and biological properties of TPSs and broaden their applications in various fields.

## Figures and Tables

**Table 1:** Influencing factors on the monosaccharide composition and molecular weight of tea polysaccharides.

Resource	Processing Technologies or Extraction Methods	Name	Monosaccharide Composition (mol.% or Mole Ratio)										M_w_/kDa	References
			Glc	Rha	Ara	Man	Rib	Xyl	Gal	Fuc	GalA	GluA		
Green tea leaves (Artificially Se-enriched Enshi)	HWE (90 °C)	ASe-TPS2	1.00	1.93	7.05			1.05			26.12		6.73	[28]
Green tea leaves (Naturally Se-enriched Enshi)		NSe-TPS2	0.10	0.28	0.59				1.00	0.07	1.24	0.49	244.32	
Green tea leaves (Se-enriched Enshi)	HWE (70 °C)	Se-TPS	0.49	0.22	0.71	0.12	0.14		1.00	0.03	1.39	0.13	1.3–1020	[32]
		Se-TPS1	0.47	0.21	0.58				1.00	0.07	1.75	0.17	110	
		Se-TPS2	0.10	0.28	0.59				1.00	0.07	1.24	0.49	240	
		Se-TPS3	0.30	0.38	0.72				1.00	0.07	0.88	0.19	250–920	
Green tea (Se-enriched Ziyang)	HWE (80 °C)	Se-TP	32.35	1.69	30.64	3.57			25.81		2.26		-	[18]
Green tea (Coarse)	WE	WE-CTPS	29.22	4.11	9.96	4.62		3.46	28.05	4.14	16.43		3.0–2560	[16]
	WAE	UAE-CTPS	36.05	2.27	9.22	4.75		5.38	27.54	6.72	8.07		3.2–3680	
	MAE	MAE-CTPS	31.09	4.03	11.84	6.17		3.64	27.06	6.84	9.33		3.1–2500	
	EE	EE-CTPS	44.24	5.40	8.86	4.38		3.15	12.32	11.78	9.87		3.0–3150	
Green tea (Low grade)	HWE	TPC	14.10	8.74	29.04	7.11		0.42	35.89	4.69			6.62, 48.5, 455	[33]
Green tea	NaCl solution extraction	TPSA	15.2	6.7	20.0	2.1	2.5	1.3	20.3				470	[34]
Green tea	BWE	GSP	7	2	19				7		65		-	[35]
Green tea (Huangshan Maofeng)	HWE (80 °C)	HMTP	7.4	2.3	28.9	4.9	6.1	1.1	35.0		11.3	3.0	-	[36]
Green tea	-	TF-1	1			3.2		1.4					231.6	[37]
		TF-2	1					1.7					46.3	
		TF-3	1		0.9			2.5					7.3	
Green tea	NaCl solution extraction	ALTPS	23.8	1.6	6.9	1.0	1.7	0.6	6.6				-	[38]
Green tea	HWE (90 °C)	TPC-W	14.10	8.74	29.04	7.11		0.42	35.89	4.69			6.62–4550	[39]
	AE	TPC-A	6.28	13.81	36.07	4.89		5.24	32.27	1.43			4.13–4940	
Green tea	HWE (70 °C)	GTPS	17	7.8	41.8	7.3		7.1	18.7				9.2–251.5	[8]
Green tea	HWE (65 °C) with EE	ATPS-2		0.68					1.00		1.58		4.43	[29]
Green tea	HWE (75 °C)	Shufeng	18.70	3.15	33.90	2.45		3.36	38.44				127	[40]
		Longjin D	17.17	3.38	32.42	2.83		2.80	41.41				106	
		Jialaoshan	16.36	3.53	32.31	3.14		2.67	42.97				121	
Green tea	HWE (75 °C)	TPF	1.01	1	18.86	5.73		2.47	18.54	1.01			-	[41]
Green tea	EE	TPS	43.27		6.49			2.60	41.11	6.53			110	[42]
Green tea leaves	BWE	7WA			1.0				0.96				71	[17]
Green tea leaves (Coarse)	Untreated	TPSU	1.00	0.88	1.19	0.34			1.00				1–330	[43]
	Extrusion treatment	TPSE4	1.00	0.12	0.93	0.13			0.62				15–330	
	Extrusion treatment	TPSE12	1.00	0.20	1.11	0.18			0.63				4–405	
Green tea leaves	Freeze-drying of TPS	TPS-F	2.82	1.0	2.77	0.45	0.58	3.12	0.14		3.88	0.26	3.3–952	[44]
	Vacuum-drying of TPS	TPS-V	5.96	1.0	2.62	0.51	0.47	1.94	0.16		3.0	0.20	3.4–910	
	Spray-drying of TPS	TPS-S	0.77	1.0	2.43	0.40	0.46	0.96	0.10		1.94	0.18	3.3–969	
	Microwave-vacuum drying of TPS	TPS-M	1.30	1.0	1.90	0.40	0.29	1.72	0.15		1.66	0.16	3.5–915	
Green tea leaves	HWE (60 °C)	TPS-1	13.6		39.4	10.2	0.3	0.5	31.0	1.3	2.1	0.9	20.8	[19]
		TPS-2	1.0	4.1	36.4	0.9		2.3	43.1	0.1	6.9	5.2	24.2	
		TPS-3	7.0	3.5	13.0	0.6	0.1	0.2	22.1	0.1	49.1	3.9	250.6	
		TPS-4	4.0	3.1	7.7	0.8	15.4	0.2	12.8	0.2	51.2	2.5	4.1, 689.1	
Green tea leaves	HWE	HWE-TLPS	1.00	4.82	0.22	0.48		0.21	2.93		2.06	0.22	1.17–413	[45]
	BWE	BWE-TLPS	1.00	0.50	1.04	0.22		0.06	1.38		1.48	0.09	1.04–458	
	EE	EE-TLPS	1.00	1.09	1.80				2.27		2.36	0.12	1.02–487	
Green tea leaves	HWE (90 °C)	TLPS	1.77	0.87	1.87	0.11	0.3	0.07	1.00	0.29	2.54	0.24	3.67–758	[10]
Green tea leaves (Xihu Longjing)	HWE (90 °C)	XTPS	5.52	9.50	8.79	3.52		1.24	13.17			11.60	1.26–810	[11]
Green tea leaves (Chawentianxia)		CTPS	11.06	8.73	11.95	3.81		1.17	16.53			15.06	12–805	
Green tea leaves (Huizhoulvcha)		HTPS	7.28	9.48	23.06	3.75		0.96	30.68			18.36	1.2–771	
Green tea leaves (Chinese tea Zhongcha 108)	Hydrothermal extraction	F_0_	7.5	33.8				2.1	13.9		1.4	41.3	51.85	[5]
		F_0.1_	22.8	46.8				3.9	26.5				40.00	
		F_0.2_	38.3	39.7					22.0				32.72	
		F_0.3_	44.7	36.4					18.9				25.27	
		F_0.4_	45.9	35.8					18.3				18.38	
White tea leaves	-	WTPS	2.2	1.1	4.2	4.5			1				29	[46]
Green tea flowers	HWE (90 °C)	TFPS	11.54	10.17	49.52	2.68		1.49	22.04	2.58			-	[47]
		TFPS-1	45.39		14.84	6.87		12.16	18.08	2.64			-	
		TFPS-2		11.19	55.16				33.65				-	
		TFPS-3		20.95	53.34				25.71				-	
Green tea flowers	HWE (80 °C)	TFPS-1	1.0	0.81				1.2	0.98				-	[9]
		TFPS-2	1.0	2.3	2.3			0.76					10.1	
Green tea flowers	HWE (90 °C)	TFPS1	1.3	1.0	2.9	0.5			3.3				500	[48]
Green tea flowers	HWE	HWE-TFPS	1.00	0.36	1.19	0.23		0.16	2.09		0.25	0.12	1.06–483	[45]
	BWE	BWE-TFPS	1.00	0.80	2.06	0.25		0.20	2.47		2.20	0.13	1.06–508	
	EE	EE-TFPS	1.00	1.33	2.90	0.21		0.24	3.20		2.10	0.12	1.18–465	
Green tea flowers	HWE (90 °C)	TFPS	0.36	0.42	0.97	0.17		0.11	1.00		0.71	0.08	2.56–1460	[10]
Green tea seeds	Na-citric acid buffer extraction	TSPS	1.95	0.35	0.95			0.15	1.00		0.23	0.07	3.66–961	[10]
Green tea seeds	Extracted with Na-citric acid buffer, enzyme and hot water in sequence	NTSPS	12.44		1.16				1				4588	[49]
		ATSPS-1	0.03	0.51	0.78	0.07		0.09	1	0.1		0.06	500	
		ATSPS-2	23.45				0.76	0.43	1				100	
Oolong tea	Ultrafiltration with M_w_ >80 kDa	OTPS1	7.90	5.50	7.31	5.78	9.43	10.32	13.11	11.85			-	[27]
	Ultrafiltration with M_w_ 30–80 kDa	OTPS2	17.13	5.39	6.90	10.90	6.90	8.43	27.32	8.20			-	
	Ultrafiltration with M_w_ 10–30 kDa	OTPS3	35.94	6.56	5.22	8.39	7.01	8.98	13.35	5.29			-	
	Ultrafiltration with M_w_ <10 kDa	OTPS4	27.86	6.13	4.99	4.16	11.79	7.39	3.65	7.35			-	
Oolong tea (Tieguanyin)	HWE (70 °C)	TTPS	26.39	5.75	26.84	2.91		0.81	35.34	1.96			25, 25, 817	[50]
Oolong tea (Fenghuangdancong)		FTPS	14.44	10.83	25.69	6.97		2.39	35.85	3.83			14, 930	
Oolong tea (Dahongpao)		DTPS	22.59	10.31	22.93	5.21		0.28	33.59	5.09			42, 110, 2640	
Oolong tea	HWE (70 °C)	OTPS	21.9	16.2	43.7				18.0				5.3–100.9	[8]
Oolong tea (Anxi Tieguanyin)	HWE (90 °C)	TTPS	12.74	7.41	13.78	5.7		1.37	20.16			15.49	1.2–762	[11]
Oolong tea	HWE (75 °C)	Fenghuangdanzong	14.38	3.65	31.70	2.60		2.79	44.87				107	[40]
		Tieguanyin	10.18	5.77	32.56	1.22		3.81	46.47				95	
Black tea	BWE	BSP	16	3					16		35		-	[35]
Black tea	HWE (70 °C)	BTPS	29.4	14.4	36.4				19.7				3.8–32.7	[8]
Dark tea (Chinese Liubao)	HWE (70 °C)	CLTPS	0.32		3.36	3.84		2.08	1.92	0.16			467, 11.4	[51]
Puerh tea	Aging time of 1 year	PTPS-1	16.52	5.34	21.86	21.59		4.04	26.93	3.64			2700	[12,52]
	Aging time of 3 years	PTPS-2	10.23	6.82	26.22	13.83		0.35	39.34	3.21			631–1930	
	Aging time of 5 years	PTPS-3	6.08	15.98	20.84	15.29		0.15	40.33	1.68			1160–3900	
Brick tea (Fuzhuan)	HWE (80 °C)	FBTPS-3		15.50	13.90	8.70			19.70		42.20		741	[53]

AE = Alkali extraction; Ara = Arabinose; BWE = Boiling water extraction; EE = Enzyme extraction; Fuc = Fucose; Glc = Glucose; Gal = Galactose; GalA = Galacturonic acid; GluA = Glucuronic acid; HWE = Hot water extraction; Man = Mannose; MAE = Microwave-assisted extraction; M_w_ = Molecular weight; Rha = Rhamnose; Rib = Ribose; TPC = Tea polysaccharide conjugates; TPS = Tea polysaccharide; UAE = Ultrasound-assisted extraction; WE = Water extraction; Xyl = Xylose.

**Table 2:** Influencing factors on chemical structures of tea polysaccharides.

Resource	Name	Isolation Methods	Structural Characterization	References
Green tea leaves (Artificial Se-enriched Enshi)	ASe-TPS2	HWE (90 °C)→Ethanol precipitation (60%)→Deproteinization→Dialysis→DEAE Sepharose fast flow gel column	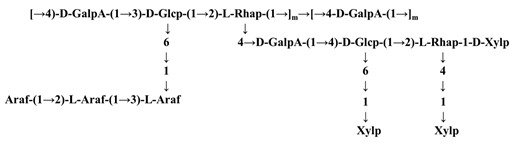	[28]
Green tea leaves (Natural Se-enriched Enshi)	NSe-TPS2		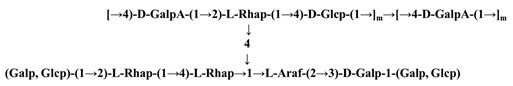	
Green tea (Zhongcha 108)	F_0.3_	WE (120 °C)→Dialysis→Deproteinization→DEAE-52 column	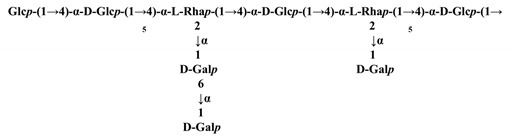	[5]
Green tea	GTPS	HWE (100 °C)→AE (10% NaOH)→Dialysis→Freeze-thawing	Main chain of (1→3)-β-Galp, substituted at O-6 by (1→6)-linked β-Galp with side chains of α-Ara*f* and terminal units of α-Rha*p*, α-Fuc*p* and α-Ara*f*	[54]
Black tea	BTPS			
Green tea	GTPI		Main chain of (1→4)-β-Xylp, substituted in O-3 by α-Araf, β-Galp and α-Glcp units	
Black tea	BTPI			
Green tea (Wufeng)	7WA	BWE (100 °C)→Ethanol precipitation (final concentration was 40% and 70%)→DEAE-cellulose column→Superdex-200 column	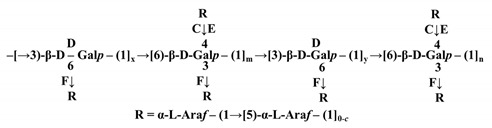	[17]
Green tea (Wufeng)	TPS1-2a TPS1-2b	BWE (100 °C)→Ethanol precipitation (final concentration was 40%)→DEAE-cellulose column→Sephacryl S-300 column	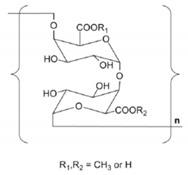	[23]
Green tea Black tea	GSP BSP	BWE (100 °C)→Ethanol precipitation (95%)→Dialysis→Freeze-thawing	Backbone with a long sequence of →4)-6-O-Me-α-D-Gal*p*A-(1→ and the side chains attached to the α-L-Rha*p* residues	[35]
Tea flowers	TFP-1	HWE (80 °C)→Ethanol precipitation (95%)→Deproteinization→Dialysis→Sephadex G-100 gel column	α-D-Gal*p*, α-L-Rha*p*, α-D-Glc*p*, α-D-GalNAc*p* and α-D-Glc*p* residues	[9]
	TFP-2		α-D-Glc*p*, α-D-Xyl*p*, α-D-GalNAc*p*, α-L-Ara*p* and α-L-Rha*p* residues	
Tea flowers	TFPS-1	BWE (90 °C)→Ethanol precipitation (95%)→Dialysis→DEAE Sepharose fast flow gel column	Backbone consisted Glu and Gal, branched chain consisted Ara, Gal and Rha	[48]
Green tea	NTPS-1	HWE (65 °C)→EE (cellulose compound enzyme)→Ethanol precipitation (75%)→Anion exchange resin D315 column →Dialysis→DEAE Sepharose fast flow gel column	β-(1→4)-linked galactopyranosyl units	[55]
Green tea	ATPS-2	EE (plant hydrolase)→HWE (60 °C)→Ethanol precipitation (95%)→Anion exchange resin D315 column→DEAE Sepharose fast flow gel column	Backbone with →4)-α-D-Gal*p*A-(1→2)-α-L-Rha*p*-(1→4)-α-D-Gal*p*A-(1→, consisting of α-1,4-D-galactopyranosyluronan and 1,2-linked rhamnosyl residues	[29]
Green tea (Wuyuan)	TGC	-	Main chain consisted of Gal, Glc and Rha by β-(1→3) linkage, while branch chains connected to main chain by β-(1→3)-, β-(1→2)- and β-(2→3)-linkages	[31]

AE = Alkali extraction; Ara*f* = Arabinofuranose; BWE = Boiling water extraction; EE = Enzyme extraction; Fuc*p* = Fuctopyranose; Gal = Galactose; GalA = Galacturonic acid; Gal*p* = Galactopyranose; Gal*p*A = Galactopyranose acid; Glc = Glucose; GlcA = Glucuronic acid; Glcp = Glucopyranose; HWE = Hot water extraction; Rha = Rhamnose; Rha*p* = Rhamnopyranose; WE = Water extraction; Xyl*p* = Xylopyranose.

## Abbreviations

AFM	Atomic force microscopy
Ara	Arabinose
BWE	Boiling water extraction
EE	Enzyme extraction
FT-IR	Fourier transform infrared spectroscopy
Fuc	Fucose
Glc	Glucose
Gal	Galactose
GalA	Galacturonic acid
GluA	Glucuronic acid
GC	Gas chromatography
GC-MS	GC-mass spectroscopy
GFC	Gel-filtration chromatography
GPC	Gel permeation chromatography
HG	Homogalacturonan
HWE	Hot water extraction
Man	Mannose
MAE	Microwave-assisted extraction
MLLS	Multiangle laser light-scattering instrument
M_w_	Molecular weight
MWD	Molecular weight distribution
NMR	Nuclear magnetic resonance spectroscopy
Rha	Rhamnose
Rib	Ribose
TFA	Trifluoroacetic acid
TPC	Tea polysaccharide conjugates
TPS	Tea polysaccharides
TWE	Traditional water extraction
TEM	Transmission electron microscopy
UAE	Ultrasound-assisted extraction
Xyl	Xylose
